# Chronic traumatic encephalopathy neuropathologic change is uncommon in men who played amateur American football

**DOI:** 10.3389/fneur.2023.1143882

**Published:** 2023-06-19

**Authors:** Grant L. Iverson, Pouya Jamshidi, Amanda O. Fisher-Hubbard, Amy Deep-Soboslay, Thomas M. Hyde, Joel E. Kleinman, Joyce L. deJong, Claire E. Shepherd, Lili-Naz Hazrati, Rudolph J. Castellani

**Affiliations:** ^1^Department of Physical Medicine and Rehabilitation, Harvard Medical School, Boston, MA, United States; ^2^Department of Physical Medicine and Rehabilitation, Spaulding Rehabilitation Hospital, Charlestown, MA, United States; ^3^Department of Physical Medicine and Rehabilitation, Schoen Adams Research Institute at Spaulding Rehabilitation, Charlestown, MA, United States; ^4^MassGeneral Hospital for Children Sports Concussion Program, Boston, MA, United States; ^5^Home Base, A Red Sox Foundation and Massachusetts General Hospital Program, Charlestown, MA, United States; ^6^Department of Pathology, Northwestern University Feinberg School of Medicine, Chicago, IL, United States; ^7^Department of Pathology, Western Michigan University Homer Stryker M.D. School of Medicine, Kalamazoo, MI, United States; ^8^Lieber Institute for Brain Development, Johns Hopkins Medical Campus, Baltimore, MD, United States; ^9^Department of Psychiatry and Behavioral Sciences, Johns Hopkins School of Medicine, Baltimore, MD, United States; ^10^Department of Neurology, Johns Hopkins School of Medicine, Baltimore, MD, United States; ^11^Neuroscience Research Australia, Randwick, NSW, Australia; ^12^School of Medical Sciences, University of New South Wales, Kensington, NSW, Australia; ^13^Department of Laboratory Medicine and Pathobiology, University of Toronto, Toronto, ON, Canada

**Keywords:** neuropathology, tau, suicide, autopsy, depression

## Abstract

**Introduction:**

We examined postmortem brain tissue from men, over the age of 50, for chronic traumatic encephalopathy neuropathologic change (CTE-NC). We hypothesized that (i) a small percentage would have CTE-NC, (ii) those who played American football during their youth would be more likely to have CTE-NC than those who did not play contact or collision sports, and (iii) there would be no association between CTE-NC and suicide as a manner of death.

**Methods:**

Brain tissue from 186 men and accompanying clinical information were obtained from the Lieber Institute for Brain Development. Manner of death was determined by a board-certified forensic pathologist. Information was obtained from next of kin telephone interviews, including medical, social, demographic, family, and psychiatric history. The 2016 and 2021 consensus definitions were used for CTE-NC. Two authors screened all cases, using liberal criteria for identifying “possible” CTE-NC, and five authors examined the 15 selected cases.

**Results:**

The median age at the time of death was 65 years (interquartile range = 57–75; range = 50–96). There were 25.8% with a history of playing American football and 36.0% who had suicide as their manner of death. No case was rated as definitively having “features” of CTE-NC by all five authors. Ten cases were rated as having features of CTE-NC by three or more authors (5.4% of the sample), including 8.3% of those with a personal history of playing American football and 3.9% of those who did not play contact or collision sports. Of those with mood disorders during life, 5.5% had features of CTE-NC compared to 6.0% of those who did not have a reported mood disorder. Of those with suicide as a manner of death, 6.0% had features of CTE-NC compared to 5.0% of those who did not have suicide as a manner of death.

**Discussion:**

We did not identify a single definitive case of CTE-NC, from the perspective of all raters, and only 5.4% of cases were identified as having possible features of CTE-NC by some raters. CTE-NC was very uncommon in men who played amateur American football, those with mood disorders during life, and those with suicide as a manner of death.

## 1. Introduction

The neuropathology of chronic traumatic encephalopathy (CTE) is currently described as a region-specific accumulation of hyperphosphorylated tau protein (p-tau) within the brain (1). The terms CTE neuropathology (2, 3) and CTE neuropathologic change (CTE-NC) (4) have been used in the literature to avoid referring to the pathology and specific clinical symptoms or disorders using the same name. CTE-NC has been reported in the brains of men who played all levels of American football (5). In one large series, this change was identified in 99% of those who played at the professional level (110/111), 91% who played at the college level (48/53), and 21% who played at the high school level (3/14) (6).

In a large-scale study by Bieniek and colleagues (7), CTE-NC (according to the 2016 consensus definition) was identified in 5.0% (15/300) of those who played sports, 1.3% who did not play sports (6/450), and 0% of women (0/273), 4.4% (21/477) of men, 7.9% of men who played American football (11/140), and 2.4% (6/245) of men who did not play sports [see the online supplementary Table 3 in Bieniek et al. (7)]. Bieniek and colleagues also characterized cases as either “positive” for CTE-NC or having “features” of CTE-NC (7), the latter meaning that some of the characteristics of CTE-NC were present without fitting *all parts of the criteria verbatim*. Features of CTE-NC were identified in 21 cases (2.8%; 21/750). They reasoned that coding cases as having features of CTE-NC was reasonable because of their limited sampling, and that more exhaustive sampling might have revealed more definitive lesions.

Other authors have recommended coding “components” of CTE-NC, not just whether it was definitively present or absent (8). Forrest and colleagues examined the prevalence of CTE-NC in brain tissue from 310 people who came to autopsy as part of an aging study and noted that 25 (8.1%) of the cases had CTE-like pathology in the depths of sulci, such as astrocytic or neuronal p-tau, but when applying strict criteria for case ascertainment they concluded that no cases met their criteria for having definitive CTE-NC—and they recommended that future studies code components of CTE-NC (8). They wrote: “to facilitate the identification of cases with the early appearance of astrocytic and/or neuronal tau pathologies, we propose the term ‘components of CTE’ (c-CTE) when reporting tau pathologies occurring at the depths of cortical sulci in higher density than observed in cortical gyri” (page 401) (8).

In 2021, the second consensus definition of CTE-NC was published (9), and the defining characteristic of CTE-NC was narrowed in an attempt to more clearly differentiate CTE-NC from age-related tau astrogliopathy (ARTAG) (10)—with more emphasis placed on p-tau aggregates in deeper cortical layers, not restricted to superficial or subpial regions. For comparative purposes, the present study used both the 2016 and the 2021 consensus definitions of CTE-NC.

### 1.1. Purpose and hypotheses

The purpose of the present study was to examine postmortem brain tissue for CTE-NC from a large sample of men, over the age of 50, most of whom had neuropsychiatric problems during life. The sample was obtained from the brain donation program at the Lieber Institute for Brain Development. This institute collaborates with medical examiner offices to obtain brain tissue from people who had neuropsychiatric problems during life to better understand the molecular mechanisms associated with these illnesses. Researchers from the institute collect detailed demographic and clinical histories from next of kin of the decedents. In a previous study using the same cases we examined the association between participation in high school football and suicide (11). There were 198 men aged 50 or older, 29.8% who participated in high school football, and 34.8% who had suicide as their manner of death. Our previous study revealed no statistically significant difference in the proportions of suicide as a manner of death among those who played football compared to those who did not play football, and those who played football were significantly less likely to have a lifetime history of a suicide attempt (11).

Using a methodology similar to Bieniek and colleagues (7), we screened tissue samples from men to determine if they had definitive CTE-NC or “features” of CTE-NC. We had three hypotheses. First, we hypothesized that, similar to prior studies using brain banks and community samples (8, 12–16), a small percentage of the cases would have evidence of CTE-NC (i.e., 10% or fewer). Second, similar to the study by Bieniek and colleagues (7), we hypothesized that those who played football would be more likely to have CTE-NC than those who did not play any contact sports. Third, we hypothesized that there would be no association between CTE-NC and suicide in this study, given the literature indicating that suicide is not likely to be a clinical feature of CTE or be associated with participation in football (11, 17–22).

## 2. Materials and methods

### 2.1. Participants

Postmortem brain tissue and accompanying information were obtained from the brain donation research program at the Lieber Institute for Brain Development (LIBD). The samples examined in this study were obtained from a collaboration between LIBD and the Office of the Medical Examiner of the Western Michigan University Homer Stryker M.D. School of Medicine (WMed), Department of Pathology (WIRB protocol #1126332). Informed consent from legal next of kin was obtained in each case at the time of autopsy. Importantly the WMed collaborative effort with LIBD includes a substantial percentage of people who completed suicide (36.0% in our study sample). Cases were identified after reviewing death scene investigations in counties subserved by the WMed Medical Examiners, typically in the 24 h preceding autopsy. The WMed catchment area at the time of case collection between 2016 and 2020 consisted of 13 counties in Western Michigan. Exclusionary criteria were as follows: not-at-fault motor vehicle accidents, homicides, suspicious deaths (e.g., elder abuse and deaths in custody), certain infectious diseases (hepatitis, human immunodeficiency virus, meningitis, sepsis, prion disease), brain-destructive processes (ischemic infarct, intrinsic brain tumors, history of neurosurgery, cerebral palsy, or remote, severe structural brain damage from trauma), encephalopathy, isolated seizure disorder (with no psychiatric history), maintenance on artificial life support for greater than 24 h, last known to be alive greater than 72 h prior, and gross evidence of autolysis or decomposition. There were 21 cases in this study that were referred at the time of organ donation *via* Gift of Life Michigan, under the same protocol, consenting procedures, and screening procedures, but did not undergo forensic examination at WMed because cause and manner of death were apparent with death investigation. Brain procurement, tissue sampling, and immunostaining were otherwise identical.

Next, of kin for potential donors who met inclusion criteria were then contacted by LIBD for consent for donation. According to previous study, 57.0% of all African–American families and 74.1% of all Caucasian families who were contacted by the LIBD agreed to donate at the time of autopsy (23). Donor history was compiled *via* medical record review, and telephone interviews with the legal next of kin (described below). Subjects were consecutively referred men aged 50 and older (i.e., 50–96) with completed immunohistochemistry performed on selected brain regions per the LIBD protocol (described below). Men were selected because the goal was to examine the association between CTE-NC and a personal history of participating in American football. Age 50 was the lower bound for inclusion in the study because the protocol at the LIBD is to conduct immunohistochemistry on tissue from people aged 50 and older. Complete clinical information, tissue samples, and immunohistochemical stains were available for 186 men.

### 2.2. Manner of death

Cases were reviewed by the medical examiner’s investigative team in a multidisciplinary morning conference prior to case selection for consent and brain procurement. Following review, manner of death (e.g., natural, accident, suicide) was determined by a board-certified forensic pathologist, in general according to guidelines from the National Association of Medical Examiners (24). The manners of death of our sample are listed in Table 1.

**Table 1:** Characteristics of the sample.

Characteristics	
Age	M = 66.2, SD = 11.3
Race (White); *n*, %	183, 98.4%
Education; *n*, %	
Fewer than 9 Years	14, 7.5%
9–11 Years	20, 10.8%
High school diploma or equivalent	69, 37.1%
Some college	38, 20.4%
Bachelor’s degree	26, 14.0%
Graduate degree	12, 6.5%
Unknown or missing	7, 3.8%
Marital status; *n*, %	
Married	89, 47.8%
Divorced	46, 24.7%
Single	28, 15.1%
Separated	4, 2.2%
Widowed	18, 9.7%
Missing	1, 0.5%
Manner of death; *n*, %	
Accident; *n*, %	57, 30.6%
Natural causes; *n*, %	56, 30.1%
Suicide; *n*, %	67, 36.0%
Undetermined, *n*, %	6, 3.2%
Depression or bipolar disorder; *n*, %, missing (*n*)	103, 55.4%
Suicide ideation; *n*, %, missing (*n*)	60, 32.3%, 28
Suicide attempts; *n*, %, missing (*n*)	49, 26.3%, 19
Family history of suicide; *n*, %, missing (*n*)	51, 27.4%, 19
Family psychiatric history; *n*, %, missing (*n*)	121, 65.1%, 23
Participation in contact/collision sports; *n*, %, missing (*n*)	58, 31.2%, 0
Participation in football; *n*, %, missing (*n*)	48, 25.8%, 0
Traumatic brain injury; *n*, %, missing (*n*)	50, 26.9%, 7

The total sample size is 186. M, mean; SD, standard deviation; *n*, sample size; NA, not applicable. For all subjects, it was known whether they were classified as having suicide as a manner of death (i.e., yes or no). For 6 people, the manner of death was not suicide, but the specific manner and cause(s) were not recorded in the database. There were 7 subjects who could not be classified on a history of traumatic brain injury (TBI) because the next of kin specified that it was “possible” that the person had a TBI but uncertain.

### 2.3. Demographic and clinical information

Initial information was obtained via a 36-item telephone interview, with the next of kin, including medical, social, demographic, family, and psychiatric history. The next of kin were also asked about a history of head trauma, personal history of concussions in sports, head injury in civilian life, and/or history that suggested severe traumatic brain injury. Although very pronounced acute structural brain injury from trauma is exclusionary for brain donation, this does not preclude consenting donors with a history of traumatic brain injury who lack obvious structural brain damage, or a history of traumatic brain injury that was not uncovered in the family interview or during the initial death scene investigation. It is also possible to uncover evidence of structural brain injury (e.g., remote contusions) that was not diagnosed during life. In our sample, 3 out of 186 cases had remote contusions detected at autopsy. Overall, a broad range of injury mechanisms (e.g., sports, car accidents, and falls from a height) and severities/types were described in the case material (e.g., skull fractures, subdural hematomas, concussion, and prolonged post-traumatic unconsciousness or coma). All levels of brain injury severity were included based on history, but for most cases there was insufficient information to classify brain injury severity more precisely. Detailed notes on traumatic brain injury were variable, and it was coded as binary.

The collective data was reviewed for retrospective clinical diagnostic interpretation, consisting of the telephone screen, autopsy results and other forensic data including toxicology, psychiatric and substance abuse treatment records, and/or general medical record reviews, and any family informant interviews that were available. All data were assembled into a comprehensive narrative summary for review by two board-certified psychiatrists. A list of lifetime diagnostic and statistical manual of mental disorders (DSM-5) psychiatric diagnoses and medical diagnoses was then compiled. LIBD also recruits control subjects, defined as subjects who were free from psychiatric and substance use diagnoses, and had negative toxicology (i.e., 15.1% of the present sample). Forensic toxicological analysis covered ethanol and volatiles, opiates, cocaine/metabolites, amphetamines, and benzodiazepines. In a subset of cases, toxicological analysis using National Medical Services, Inc., was obtained, including nicotine/cotinine testing, cannabis testing, and an expanded forensic panel to cover any substances not otherwise tested including prescription medications.

### 2.4. History of playing sports

The next of kin informants were also asked about decedents’ history of engagement in contact or collision sports, such as boxing, football, and rugby, and this variable was coded as yes or no. Study subjects were categorized into groups based on whether they played contact sports in general, played American football specifically, or played no contact or collision sports. The informants reported that 48 men played high school football and 4 played college football. Six other subjects were identified as playing football, but their level was not clear. Hockey was played by three men, and two men played both hockey and football. Three men were noted to have participated in wrestling. Three men participated in kickboxing or boxing, and two men participated in either auto racing or motorcycle racing with multiple possible concussions (head injuries) noted by the persons’ next of kin.

### 2.5. Brain sampling and immunohistochemistry methodology

A minimum of seven brain samples per donor were obtained for histopathology: right middle frontal gyrus (Brodmann area 8), right superior/middle temporal cortex (Brodmann area 22), right inferior parietal lobule (Brodmann area 40), right occipital calcarine cortex (Brodmann area 17), hippocampus at the level of the lateral geniculate nucleus (including the contiguous parahippocampal gyrus and inferior temporal neocortex in most cases), midbrain, and cerebellar hemisphere with dentate nucleus (Table 2). The sampling list reflects the Lieber protocol for all cases. It contains fewer brain regions than the sampling protocol recommended in consensus articles (1, 9), but is more extensive than other large studies reported in the literature (7, 8). Neuropathology assistants were instructed to obtain cortical samples that included sulcal depths, although in a small minority of sections, a sulcal depth was difficult to identify histologically. Repeat sampling was not pursued in those instances because the remaining tissue was frozen and reserved for genetic studies, per the purpose of the Lieber brain collection.

**Table 2:** Sampling and staining protocol.

Region	Immunostains performed	
Right middle frontal gyrus	p-tau	Aβ
Superior/middle temporal gyrus	p-tau	Aβ
Inferior parietal lobule	p-tau	Aβ
Calcarine cortex	p-tau	Aβ
Hippocampus	p-tau	—

p-tau, phosphorylated tau, using monoclonal antibody AT8 as the primary antibody; Aβ, amyloid-β immunohistochemistry, using monoclonal antibody BAM01 as the primary antibody.

Tissue samples were fixed in 10% neutral buffered formalin, processed through graded ethanol and xylene solutions, and embedded in paraffin. Five μm-thick sections were prepared from all samples and stained with hematoxylin and eosin for routine histopathology. Five μm-thick sections of the cortical samples and hippocampus were prepared for p-tau immunohistochemistry, along with 5 μm thick sections of the cortical samples for amyloid-β (Aβ) immunohistochemistry (Table 3), which is only performed on decedents age 50 and older, per Lieber protocol. All immunohistochemical stains were performed using an automated immunostainer and antigen retrieval, along with positive and negative (omission of primary antibody) controls. Antibodies included p-tau (AT8, ThermoFisher, 1:250 dilution) and Aβ (BAM01, ThermoFisher, 1:50 dilution). P-tau preparations were pretreated with heat-induced citrate for 20 min. Aβ preparations were pretreated with formic acid for 30 min.

**Table 3:** Antibodies and conditions.

Antibody	Clone	Vendor	Dilution	Antigen retrieval
Phospho-tau	AT8	Thermo Scientific	1:250	ThermoScientific Dewax and HIER L Buffer, 20 min., 98°C
Amyloid-β	BAM01	Thermo Scientific	1:50	95% formic acid, 30 min

All slides were scanned into virtual microscopy at 20× magnification. They were examined using Aperio ImageScope Pathology View software.

The histopathological analyses were customized for the case material obtained by the Lieber Institute for Brain Development, which focuses on neuropsychiatric disorders and generally avoids cases with confounding structural pathology or neurodegenerative diseases. The analyses are therefore a screening process for age-related proteinopathy or early/preclinical neurodegenerative disease, and they are abbreviated in comparison with recommended methodology for Alzheimer’s disease neuropathologic change (ADNC) (25). They are also abbreviated relative to recommendations for CTE-NC from the 2016 consensus meeting (1), and CTE-NC from a 2021 consensus meeting (9), which are intended as a comprehensive screen for the lesion of interest. Other studies involving brain banks have used sampling that is less than recommended by the consensus groups (7, 8, 13, 26, 27).

P-tau and amyloid-β were semi-quantitated using modifications of Braak (28) and CERAD (29), which we used in a prior study (30). Of note, these modifications are not meant to approximate or duplicate either methodology, but to provide a semi-quantitative examination of proteinopathy with the available samples. Briefly, p-tau involvement of the parahippocampal gyrus constitutes modified Braak stage I-II (B1), p-tau involvement of the hippocampal pyramidal layer constitutes modified Braak stages III and IV (B2), and p-tau involvement of the neocortex constitutes modified Braak stages V and VI (B3). “Modified CERAD” involves semi-quantitating Aβ (rather than thioflavin-S or Bielschowsky silver impregnation) into sparse, moderate, or frequent Aβ plaques according to the schematic representations in the CERAD article, recognizing that this modification is an assessment of Aβ plaques in the cortical samples, rather than an assessment of neuritic plaques *per se*. This is an appropriate modification with increased sensitivity of detection of age-related Aβ deposits, given the Lieber Institute’s focus on psychiatric diseases rather than Alzheimer’s disease.

### 2.6. Approach to identifying CTE-NC and features of CTE-NC

We defined the presence of CTE-NC using both the 2016 and the 2021 consensus definitions. Using the first consensus definition of CTE-NC (1), the operational definition used by Bieniek and colleagues was as follows (7): “cases with pathology consistent with all aspects of the CTE neuropathology consensus criteria, namely (A) ‘hyperphosphorylated tau aggregates,’ (B) ‘in neurons, astrocytes, and cell processes,’ (C) ‘around small vessels’ and (D) ‘in an irregular pattern at the depths of the cortical sulci,’ were classified as ‘CTE-positive’ (the 2016 consensus criteria were cited). ‘Features of CTE’ was used to designate cases with multiple lesions suggestive of CTE pathology without fitting all parts of the criteria verbatim, exclusive of Alzheimer-type pathology and aging-related tau astrogliopathy” (page 66) (7). The exact wording of the pathognomonic feature required for diagnosis, according to the first consensus statement, is as follows (1): “the pathognomonic lesion consists of p-tau aggregates in neurons, astrocytes, and cell processes around small vessels in an irregular pattern at the depths of the cortical sulci” (page 81). We used that definition in the present study, which was applied by all neuropathology raters to the samples that were selected after screening.

In 2021, the second consensus definition of CTE-NC was published, and the defining pathognomonic characteristic of CTE-NC changed with a particular emphasis on trying to disambiguate CTE-NC from ARTAG (9)—especially by removing subpial astrocytic p-tau as a supportive feature and making glial p-tau (e.g., thorn-shaped astrocytes; TSA) in the depth of a sulcus not necessary for the definition. For the second consensus conference, CTE-NC was defined as: “a pathognomonic lesion consists of p-tau aggregates in neurons, with or without glial p-tau in TSA, at the depth of a cortical sulcus around a small blood vessel, in deeper cortical layers not restricted to subpial and superficial region of the sulcus” (page 215). We also used that definition in the present study.

Features of CTE-NC were assessed, meaning that some of the characteristics of CTE-NC were present but the case did not meet strict criteria for CTE-NC. These include, for example, tau-positive astrocytes, neurons, or neurites concentrated around a perivascular space but not clearly in the sulcal depth, or tau-positive astrocytes, neurons, or neurites in a patchy distribution at the sulcal depth but not clearly concentrated around a blood vessel.

Interpreting CTE-NC in the presence of aging-related pathologies and Alzheimer’s disease changes is challenging. The authors of the 2021 consensus criteria, in the limitations section of their article, wrote: “the study cases were relatively “pure” CTE (with the exception of some AD pathology) despite reports indicating that comorbid neurodegeneration (AD, amyotrophic lateral sclerosis, frontotemporal lobar degeneration, Lewy body disease, multiple system atrophy, etc.) is common in older individuals with CTE (4, 5, 12, 31, 32). Distinguishing CTE from concomitant neurodegenerative and aging-related pathologies represents a topic of interest for future studies, and the consensus committee makes no assertions regarding CTE in the presence of AD or other neurodegenerative disorders at this time” (page 218) (9). The present study focused on the definitions as proposed and depicted in the consensus articles, recognizing the limitations of the current CTE-NC assessment methodology, as other research groups have.

Two authors (PJ, RC) blinded to all clinical and demographic information (including age) screened all the cases, with the purpose of selecting cases for *possible* CTE-NC for review by the expanded panel of neuropathology experts. These two authors used the following guidelines for screening for possible CTE-NC: Patchy p-tau aggregates apparent at medium or low magnification either around blood vessels, and/or at or near a sulcal depth, that could reasonably raise the possibility of CTE-NC. The two reviewers worked independently. If either reviewer identified the case as having possible features of CTE-NC, that case was included in the sample for all reviewers to examine. Five authors from three countries and four academic institutions independently examined, or re-examined, the cases identified through the initial screening process (PJ, RC, AF-H, L-NH, CS).

### 2.7. Statistical analyses

The proportions were computed and compared using *χ^2^* tests. Using G*Power 3.1.9.7 for *z* tests and differences between two independent proportions, assuming an allocation ratio of 3 (i.e., approximately 25% of the sample played American football) and assuming that CTE-NC would be identified in 10% of those who played football and 1% of those who did not, using a one-tailed directional test, with a critical *z* = −1.645 (alpha 0.05) and power = 0.80, a total sample size of 175 would be required to detect this 9% effect size difference in proportions (i.e., 44 former football players and 131 who did not play football). Using the same parameters (allocation ratio = 3, alpha = 0.05, power = 0.80), but assuming that CTE-NC would be detected in 5% of those who did not play football and 15% of those who did play football, detecting that difference in proportions would require a sample size of 269 (67 former football players and 202 who did not play football). Mann Whitney U tests were used to compare groups based on age. All statistical analyses were conducted using IBM SPSS Statistics 26.

## 3. Results

The sample included 186 men. The median age of the men at the time of death was 65 years [M = 66.2, SD = 11.3, interquartile range (IQR) = 57–75; range = 50–96]. One-quarter of the sample had a history of playing football (25.8%), one-quarter had a history of TBI (26.9%), half had a history of a mood disorder during life (55.4%), and approximately one-third of the sample had suicide as their manner of death (36.0%). The characteristics of the sample are presented in Table 1.

### 3.1. Initial screening for features of CTE-NC

Of the 186 men, 15 initially screened positively for review by the expanded panel of authors with expertise in neuropathology, representing 8.1% of the total sample. This initial screening was designed to be overly inclusive to identify cases that could then be reviewed by more authors. For the purposes of this study, the presence of ADNC (i.e., neuritic plaques) was not *a priori* exclusionary for screening or for the assessment of “features of CTE-NC.”

### 3.2. Cases with features of CTE-NC

**Figure 1:**
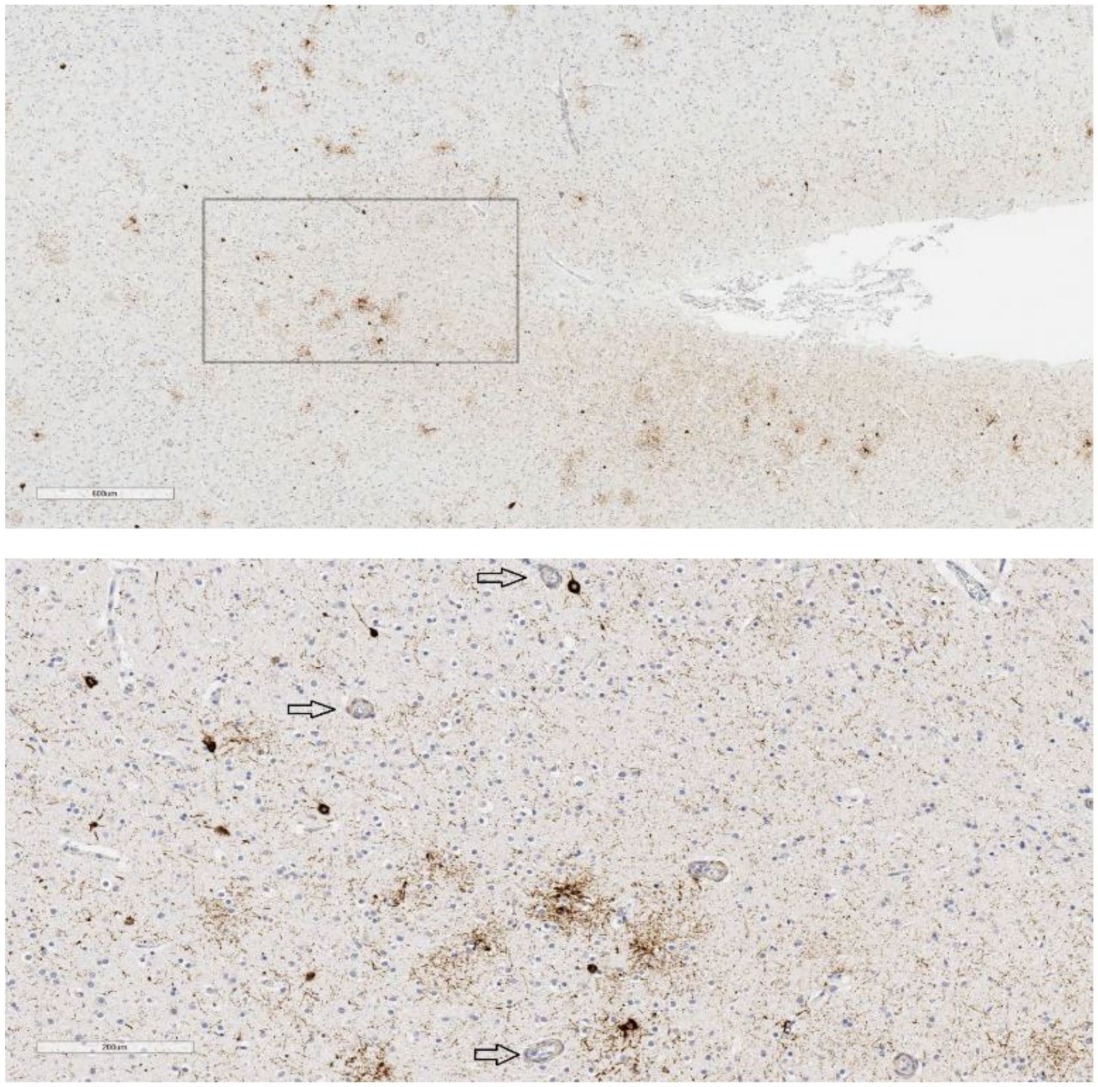
P-tau in temporal lobe sulcus. Case 9, age 83, suicide = yes, mood disorder = yes, traumatic brain injury = no, football = no, contact sports = no. P-tau aggregates are seen in the bank of this temporal lobe sulcus in a somewhat patchy distribution, not restricted to superficial or subpial regions (upper image scale bar = 600 μm). The neuronal and astrocytic involvement is in the vicinity of small blood vessels (arrows) but did not meet all criteria for definitive CTE-NC (lower image scale bar = 200 μm; from the box in the upper image).

**Figure 2:**
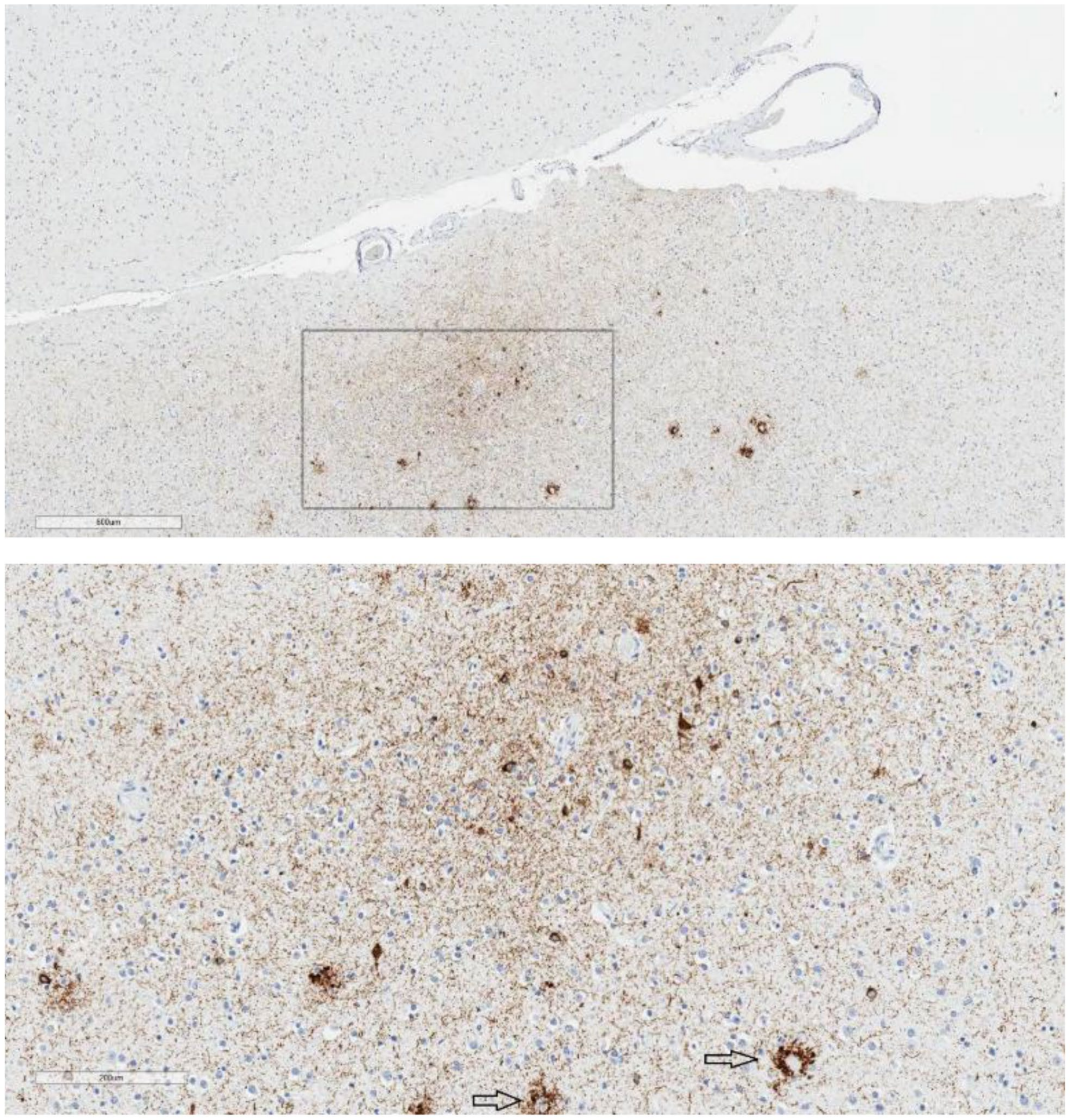
Epicenter of p-tau in the parietal cortex. Case 1, age 91, suicide = no, mood disorder = no, traumatic brain injury = no, football = yes. Low magnification shows an “epicenter” of p-tau immunolabeling in the parietal cortex, not restricted to the superficial or subpial region (upper image; scale bar = 600 μm). At high magnification (lower image; scale bar = 200 μm), the immunolabeling consists of neuronal p-tau, perivascular p-tau, and an abundance of neuropil threads. The focus appears to be in the ‘bank’ of the sulcus rather than at the extremity of the sulcal depth. There is also p-tau pathology associated with neuritic plaques (arrows). This was considered CTE-NC-like by some reviewers but was not considered definitive for CTE-NC by all the reviewers.

Those 15 cases were then examined, or re-examined, by five authors (PJ, CS, L-NH, AF-H, RC), also blinded to all clinical and demographic information. No case was identified as definitively meeting criteria for CTE-NC based on the 2016 or 2021 definition by the authors. No case was rated as having “features” of CTE-NC, based on the 2016 or the 2021 definition, unanimously by all five reviewing authors (PJ, CS, L-NH, AF-H, RC). There were two cases that were rated by four authors (out of the five) as having features of the 2016 definition and one case that was rated by four authors as having features of the 2021 definition. Cases rated as “possible,” or having “features,” by three or more authors were selected for further analysis. Using these criteria, there were six cases (3.2%) that had features according to the 2016 definition and eight cases (4.3%) that had features according to the 2021 definition, and a total of 10 cases that had features of one or both of the definitions (5.4%). Examples of neuropathology visualized in these cases are provided in Figures 1, 2.

Those 10 cases who screened positively for features of CTE-NC (M = 73.9 years, Md = 76, SD = 16.1, Interquartile range [IQR] = 53.5–89.5) were a median of 11.5 years older than those who did not have features of CTE-NC (M = 65.8, Md = 64.5, SD = 10.9, IQR = 57–73.8), although this difference was not statistically significant (*U* = 1,161, *p* = 0.09). There was no significant association between a personal history of playing American football [American football = 8.3%, no contact sports = 3.9%; *χ^2^*(1, 176) = 1.410, *p* = 0.235] or contact sports broadly (including American football) [contact sports = 8.6%, no contact sports = 3.9%; *χ^2^*(1, 186) = 1.744, *p* = 0.187] and screening positively for features of CTE-NC. There was no significant association between having a history of TBI and screening positively for features of CTE-NC [TBI = 4.0%, no TBI = 6.2%; *χ^2^*(1, 179) = 0.331, *p* = 0.565]. There was no significant association between having a mood disorder and screening positively for features of CTE-NC [mood disorder = 5.5%, no mood disorder = 6.0%; *χ^2^*(1, 186) = 0.124, *p* = 0.725]. There was no significant association between suicide as a manner of death and screening positively for features of CTE-NC [suicide = 6.0%, not suicide = 5.0%; *χ^2^*(1, 186) = 0.073, *p* = 0.788].

The neuropathology characteristics of these 10 cases is presented in Table 4. Most (8/10) had modified Braak scores between III and V. Neuritic plaques were identified by p-tau immunostaining in 5 cases, and 2 of those cases were rated as having “frequent” amyloid-β by the modified CERAD protocol. Superficial cortical p-tau-positive neurofibrillary tangles, in the absence of obvious amyloid beta in the same region, was present in most cases. In the hippocampus, preferential CA2 p-tau was present in 8 cases, and CA3/4 pretangles and prominent dendritic swellings were present in 8 cases. Seven cases had some form of ARTAG, with subpial ARTAG being present in 3 cases.

**Table 4:** Neuropathology identified in the 10 cases identified as having features of CTE-NC.

	Demographic and Clinical Features					Neuronal tau		ARTAG			Hippocampal tau		Amyloid	
Case	Age	Sport	TBI	Mood disorder	Suicide	Modified Braak	Superficial cortical NFT	1	2	3	CA2	CA3/4	Neuritic plaques	Modified CERAD
1	91	Football	No^a^	No	No	B3	No	No	No	No	Yes	Yes	Yes	3
2	92	None	No	No	No	B3	No	No	No	No	Yes	Yes	Yes	2
3	77	Football	No	No	No	B2	Yes	No	No	No	Yes	Yes	No	2
4	75	Football^b^	Yes	No	No	B2	Yes	No	No	Yes	Yes	Yes	Yes	2
5	75	None	No	No	No	B2	No	Yes	Yes	Yes	Yes	Yes	Yes	3
6	54	None	Yes	Yes	Yes	B1	Yes	Yes	No	Yes	No	No	No	0
7	52	Football	No	Yes	No	B1	Yes	No	No	Yes	No	No	No	1
8	51	Hockey	No	Yes	Yes	B2	Yes	No	Yes	Yes	Yes	Yes	No	0
9	83	None	No	Yes	Yes	B2	Yes	Yes	Yes	Yes	Yes	Yes	No	1
10	89	None	No	Yes	Yes	B2	Yes	No	No	Yes	Yes	Yes	Yes	2

Age at time of death. Case 4 also played football at the college level. TBI, traumatic brain injury.

a This subject experienced head, neck, and spine injuries as the cause of death but did not have a known history of TBI prior.

b He played both high school and college football. For age-related tau astrogliopathy (ARTAG), 1 = subpial, 2 = subependymal, and 3 = cortical. For CA2, yes means there was preferential p-tau in the CA2 region. For CA3/4, yes means there were pretangles and prominent dendritic swellings.

## 4. Discussion

The most notable finding in this study was the very low proportion of cases with CTE-NC identified in this large sample of men, over the age of 50, some of whom played amateur American football and many of whom had serious psychiatric disorders during life. Although presence of p-tau aggregates in the samples was nearly universal, we did not identify a single definitive case of CTE-NC, from the perspective of all raters. In addition, the raters did not unanimously agree on any single case having features of CTE-NC. There were only 10 cases, 5.4% of the total sample, that three or more of the five raters considered to have some feature of the pathognomonic lesion. The results of this study are fairly similar to the results from Bieniek and colleagues (7), who found that only 4.4% (21/477) of the men in their sample had evidence of CTE-NC and an additional 4.2% (20/477) had features of CTE-NC.

In the Bieniek et al. study, those who were CTE-NC positive were, on average, 10 years older at the time of death—which was similar to the age difference between groups in the present study. In the Bieniek et al. study, 29% of the men had a history of playing football and 7.9% were rated CTE-NC positive and 7.1% were considered to have features of CTE-NC (per Table 3 of their online Supplementary material). In the present study, 25.8% of the men had a history of playing football, none were considered definitively CTE-NC positive, and 8.3% had features of CTE-NC. Bieniek and colleagues reported that men who had features of CTE-NC, compared to those who did not, had a significantly lower likelihood of anxiety, a higher likelihood of dementia, movement disorders, psychosis, and ADNC, and they did not significantly differ on depression or suicide as a manner of death. In the present study, there were no statistically significant associations between having a feature of CTE-NC and a personal history of playing football, playing contact sports including football, a history of TBI, having a mood disorder, or having suicide as a manner of death. Our study was sufficiently powered to detect effect sizes that are small-medium or larger in magnitude using chi-square tests. Consequently, results of our chi-square tests may not detect a statistically significant difference when a small effect size is present due to sample size limitations. Moreover, given that the frequency of features of CTE-NC in the present study was so small, that further limits the ability to detect small differences between groups.

CTE-NC, CTE-like pathology, or features of CTE-NC have been identified in people from the general population (14, 16, 26, 33, 34) and in people with (i) schizophrenia who have undergone leukotomy (35), (ii) temporal lobe epilepsy (36), (iii) amyotrophic lateral sclerosis (37, 38), (iv) multiple system atrophy (12), and (v) other neurodegenerative diseases (13). Some of the cases in the aforementioned studies have no known participation in collision or contact sports and no known exposure to repetitive neurotrauma, thereby complicating clinicopathological studies aimed at identifying thresholds of exposure for at risk individuals. Exposure to contact and collision sports should be studied in large population-based cohorts to refine our understanding of the association between repetitive hits to the head and the etiology of CTE-NC.

Recently published large studies from the Sydney Brain Bank in Australia (*N* = 636) (16) and a community sample in the United States (*N* = 532) (14) have identified extremely few cases of CTE-NC (less than 1%) and often in elderly individuals, consistent with the current study. In a recent study of 225 former military personnel, CTE-NC was identified infrequently, in 4.4%, and it usually involved minimal neuropathologic changes—with half of the identified cases having only a single pathognomonic lesion (15). A large European study (*N* = 310) reported no cases of CTE-NC (8) and in a study of 180 consecutive autopsies in Australia, only four cases of low-stage CTE-NC were identified (2.2%) (34). These large studies suggest that CTE-NC might be uncommon or rare in the general population, although we currently have no clear estimates of the prevalence of CTE-NC in a representative sample of the broader general population and there are no longitudinal case control or cohort studies of CTE-NC that allow us to examine relative risk in a population of people who have been exposed, versus not exposed, to concussions and repetitive hits to the head.

We hypothesized that there would be no association between CTE-NC and suicide in this study, given the literature indicating that suicide is not likely to be a clinical feature of CTE or be associated with participation in football (11, 17–22). Moreover, suicidality during life and suicide as a manner of death are extraordinarily complex, multifactorial in causation, and difficult to predict in individuals, families, and communities. Risks factors for suicidality extend back to childhood in some people, such as experiencing abuse and neglect (39, 40). Men are more likely to complete suicide than women (41), and for people who are experiencing depression additional risk factors for suicide include a family history of a mental health problems, prior suicide attempts, experiencing hopelessness, experiencing anxiety, and misusing alcohol or drugs (42). Some precipitating events for suicide include a life crisis, intimate partner problems, health problems, and occupational, financial, or legal problems (41). In older adults, suicidality is associated with functional disability and a diverse range of medical problems (43). Using this same sample, we previously reported no association between suicide and participation in high school football (11) and the present study did not find an association between suicide and CTE-NC or features of CTE-NC. Although this sample is not representative of the general population, over one-third of donors had suicide as their manner of death making this one of the largest studies to investigate associations between football, CTE-NC, and suicide.

### 4.1. Study strengths

This study has several unique features and strengths. First, the brain bank is different from all prior brain bank studies in that it specializes in donations from people who suffered from psychiatric and neuropsychiatric disorders. The sample size was larger than many prior studies (5, 12, 26, 44, 45), the proportions with severe psychiatric disorders and suicide as their manner of death were quite high, and the donations were sought without regard to contact sport history. Second, next of kin were carefully interviewed by a board-certified neurologist or psychiatrist to document the decedents psychiatric history. Finally, two specialists in neuropathology blindly screened all cases, and then five experts in neuropathology screened the cases that were selected for further review. The experts in neuropathology came from three different countries (United States, Canada, and Australia) and four different academic institutions—which promotes confidence that the central finding, CTE-NC is very uncommon in this sample of men, is accurate.

### 4.2. Study limitations

This study has important conceptual limitations. First, the exposure variable was based on information obtained from the next of kin, and it was binary. Similar to most previously published postmortem studies, we could not document, classify, quantify, or analyze number of years of exposure to football or contact sports. Second, although our sample size was larger than some prior studies, and sufficiently powered to detect small-medium effect sizes, our pathology was so uncommon that we were underpowered to detect small group differences. Third, our pathology sampling was less than what is recommended by the two consensus conference statements (1, 9), although more extensive than other large studies as noted above (7, 8). The consensus criteria recommend sampling more brain regions and the authors state that if CTE-NC is not found when it is suspected, additional sampling is recommended. If searching for the pathognomonic lesion in a case that was initially negative for CTE-NC, the authors wrote: “it is recommended that the resampled tissue capture 4–8 cortical sulci (preferably bilateral) from the superior frontal, dorsolateral superior frontal, superior middle temporal, and/or inferior temporal gyri” (page 216). With more extensive sampling, we might have identified more evidence of CTE-NC. Fourth, TBI history was obtained through telephone interview with next of kin and medical record review. It was not further refined or subclassified as might be performed in epidemiological studies examining TBI *per se*. Finally, this study suggests that CTE-NC is uncommon in men who played high school American football, a substantial percentage of whom had serious psychiatric and substance misuse problems. However, data derived from an autopsy sample that requires tissue donation and consent from legal next of kin have inherent bias in acquisition. The present sample, without question, has a major inherent bias because the research program specializes in donations from people who suffered from psychiatric and neuropsychiatric disorders—this sample clearly is not representative of men from the general population. This is a sample of convenience, not a cohort or case–control study that can be used to examine prevalence or to examine issues relating to causation.

### 4.3. Disentangling CTE-NC from age-related tau

Neuropathologists have long recognized that neurofibrillary tangles and p-tau aggregates occur with age (46–50) and, by themselves, they are not necessarily indicative of a clinical disease. Similarly, p-tau-positive glia are common in aging and in specific neurodegenerative diseases (51–54). Age-related tau pathology was common in our sample. For example, seven of our 10 cases had some form of ARTAG, with subpial ARTAG being present in 3 cases. ARTAG frequently co-occurs with CTE-NC (55–57). Between 2009 and 2021, ARTAG neuropathology in subpial, subependymal, and perivascular locations was described unambiguously as *characteristic of CTE-NC* or as *a supportive feature of CTE-NC* (5, 58). In 2009, in their seminal review, McKee and colleagues included ARTAG-like features in their description of the pathology (58), and it was also included as part of the definition of CTE-NC in their large case series published in 2013 (5). In 2016, Kovacs and colleagues noted that ARTAG has features that overlap those of CTE-NC (10). In 2016, the first consensus definition of CTE-NC included subpial astrocytic tau as a “supportive feature” (1). Goldfinger and colleagues speculated that the co-occurrence ARTAG and CTE-NC raises the question whether they are, in fact, clearly distinct entities versus being on a common spectrum (55). Moreover, in 2020 researchers reported that the distribution of astroglial pathology in the depths of cortical sulci, alone, and not neuronal pathology, might be more reflective of CTE-NC (56). A recently published study reported that CTE-NC is more likely to be accurately identified when both neuronal and astroglial tau pathologies can be visualized (57). Nonetheless, subpial ARTAG is no longer considered to be part of the 2021 consensus definition of CTE-NC (9), and we did not consider it necessary or sufficient for identifying CTE-NC.

Prior to the first consensus conference, features of PART (for example, p-tau in the medial temporal lobe in the absence of Aβ) appeared to overlap with CTE-NC (5, 58, 59). The first consensus conference definition of CTE-NC included neuronal p-tau in the CA2 region of the hippocampus, hypothalamus, thalamus, and amygdala and defined that pathology as “supportive features” of CTE-NC (1). PART is characterized by neuronal p-tau in the medial temporal lobe, olfactory areas (bulb and cortex), basal forebrain, deep gray matter, and brainstem with sparse or absent Aβ. Therefore, *both* the presence of p-tau and the lack of Aβ define PART and distinguish PART from ADNC (60). PART is usually considered to be a pathological finding in older adults, but it has been reported in adults at earlier ages (61). Like CTE-NC, PART is at present a neuropathological entity; it has not been defined as a canonical neurodegenerative disease. In the 2021 consensus criteria, the authors continued to grapple with differentiating PART from CTE-NC. Specifically, they wrote: “after panel discussion, the group determined that if a case is diagnosed as CTE based on cortical pathology, the presence of NFT in the medial temporal lobe is considered a feature of CTE and an additional diagnosis of PART is not warranted” (page 217) (9). Moreover, neuronal p-tau in the entorhinal cortex and the hippocampus, which is characteristic of PART, is used to “grade the severity” of CTE-NC according to the 2021 consensus criteria. Some might consider this to propose a metaphysical dilemma, or arguably a paradox, whereby if a single pathognomonic lesion is identified in the neocortex, then age-related accumulations of neuronal tau become transmuted into CTE-NC. Stated differently, PART is PART, unless and until a single pathognomonic CTE-NC lesion is identified—and then the tau pathology of PART becomes the tau pathology of CTE-NC. The research community is encouraged to continue to grapple with disentangling age-related tau pathology and disease-related tau pathology from the neuropathology believed to be unique to, and etiologically related to, CTE-NC.

### 4.4. Conclusion

Large studies from Australia (16), the United States (14), and Austria (8) suggest that CTE-NC might be uncommon or rare in the general population—at least as reflected by donations to brain banks. Some studies in the United States suggest that CTE-NC is very uncommon in former amateur contact and collision sport athletes (7) and former military personnel (15). The present study was designed to examine CTE-NC, or features of CTE-NC, in former high school football players, similar to the study of Bieniek and colleagues (7). Bieniek and colleagues concluded that participation in football, specifically beyond the high school level (i.e., at the college level), was associated with greater odds of having CTE-NC (approximately 90% of their sample played at the high school level and 10% also played at the college level). We did not have reliable information relating to how many in our sample played football at the college level, although four cases were identified by the next of kin as having played in college. We did not identify a single definitive case of CTE-NC, in our sample, and only 5.4% of cases were identified as having possible features of CTE-NC by some raters. In the present study, CTE-NC was very uncommon in men who played amateur football, those with mood disorders during life, and those with suicide as a manner of death. Future research is needed to determine whether CTE-NC underlies a distinct clinical disease, whether it reflects accelerated brain aging, and how common it is in people with varying degrees of exposure to repetitive neurotrauma.

## Data availability statement

The original contributions presented in the study are included in the article/Supplementary material, further inquiries can be directed to the corresponding author.

## Author contributions

GI conceptualized the study, conducted the literature review, conceptualized and conducted the statistical analyses, wrote sections of the manuscript, and agrees to be accountable for the content of the work. PJ conducted the initial neuropathology screening of the entire sample, conducted in-depth analysis of the selected cases, edited versions of the manuscript, and agrees to be accountable for the content of the work. AF-H conducted in-depth neuropathological analysis of the selected cases, edited drafts of the manuscript, and agrees to be accountable for the content of the work. AD-S completed team-based clinical diagnostic reviews for each case, managed the brain bank data, participated in conceptualizing the study, reviewed drafts of the manuscript, and agrees to be accountable for the content of the work. TH administratively manages the brain bank, conducted interviews with next of kin, completed team-based clinical diagnostic reviews for each case, participated in conceptualizing the study, reviewed drafts of the manuscript, and agrees to be accountable for the content of the work. JK administratively manages the brain bank, conducted interviews with next of kin, completed team-based clinical diagnostic reviews for each case, participated in conceptualizing the study, reviewed drafts of the manuscript, and agrees to be accountable for the content of the work. JJ serves as the site principal investigator for the brain donation program, wrote portions of the manuscript, edited drafts, and agrees to be accountable for the content of the work. CS conducted in-depth neuropathological analysis of the selected cases, edited drafts of the manuscript, and agrees to be accountable for the content of the work. L-NH conducted in-depth neuropathological analysis of the selected cases, edited drafts of the manuscript, and agrees to be accountable for the content of the work. RC assisted with conceptualizing the study, assisted with the literature review, conducted the initial neuropathology screening of the entire sample, conducted in-depth analysis of the selected cases, wrote portions of the manuscript, edited drafts of the manuscript, and agrees to be accountable for the content of the work. All authors contributed to the article and approved the submitted version.

## Funding

GI acknowledges unrestricted philanthropic support from ImPACT Applications, Inc., the Mooney-Reed Charitable Foundation, the National Rugby League, and the Schoen Adams Research Institute at Spaulding Rehabilitation. He has received research funding from the Wounded Warrior Project™ to conduct research relating to CTE and traumatic encephalopathy syndrome, but not for this study. The above entities were not involved in the study design, collection, analysis, interpretation of data, the writing of this article, or the decision to submit it for publication.

## Conflict of interest

GI serves as a scientific advisor for NanoDX®, Sway Operations, LLC, and Highmark, Inc. He has a clinical and consulting practice in forensic neuropsychology, including expert testimony, involving individuals who have sustained mild TBIs (including former athletes), and on the topic of suicide. He has received research funding from several test publishing companies, including ImPACT Applications, Inc., CNS Vital Signs, and Psychological Assessment Resources (PAR, Inc.). He has received research funding as a principal investigator from the National Football League, and subcontract grant funding as a collaborator from the Harvard Integrated Program to Protect and Improve the Health of National Football League Players Association Members.

RC is subcontracted to the Lieber Institute for Brain Development to assist with brain examinations. He is a consultant on a grant from the National Football League. He has a consulting practice in forensic neuropathology, including expert testimony, some of which involves former contact sport athletes.

The remaining authors declare that the research was conducted in the absence of any commercial or financial relationships that could be construed as a potential conflict of interest.

## Publisher’s note

All claims expressed in this article are solely those of the authors and do not necessarily represent those of their affiliated organizations, or those of the publisher, the editors and the reviewers. Any product that may be evaluated in this article, or claim that may be made by its manufacturer, is not guaranteed or endorsed by the publisher.
