# Supplementary material for: Chronic traumatic encephalopathy neuropathologic change is uncommon in men who played amateur American football
Source: Front Neurol. 2023 Jun 19;14:1143882. doi: 10.3389/fneur.2023.1143882 (PMC10315537; doi:10.3389/fneur.2023.1143882)
Supplement: Supplementary file 1 [file Data_Sheet_1.pdf]

Online Supplementary Material

Frontiers in Neurology, Neurotrauma Section

Chronic Traumatic Encephalopathy Neuropathologic Change is Uncommon in Men who Played Amateur American Football

Grant L. Iverson, Ph.D.[1-5*], Pouya Jamshidi, M.D.[6], Amanda O. Fisher-Hubbard, M.D.[7], Amy Deep-Soboslay, M.Ed.[8], Thomas M. Hyde, M.D., Ph.D.[8,9,10], Joel E. Kleinman, M.D., Ph.D.[8,9], Joyce L. deJong, D.O.[7], Claire E. Shepherd, Ph.D.[11,12], Lili-Naz Hazrati, M.D., Ph.D.[13], Rudolph J. Castellani, M.D.[6]
[1]Department of Physical Medicine and Rehabilitation, Harvard Medical School, Boston, Massachusetts, USA.
[2]Department of Physical Medicine and Rehabilitation, Spaulding Rehabilitation Hospital, Charlestown, Massachusetts, USA
[3]Department of Physical Medicine and Rehabilitation, Schoen Adams Research Institute at Spaulding Rehabilitation, Charlestown, Massachusetts, USA
[4]MassGeneral Hospital for Children Sports Concussion Program, Boston, MA, USA
[5]Home Base, A Red Sox Foundation and Massachusetts General Hospital Program, Charlestown, Massachusetts, USA
[6]Department of Pathology, Northwestern University Feinberg School of Medicine, Chicago, Illinois, USA
[7]Department of Pathology, Western Michigan University Homer Stryker M.D. School of Medicine, Kalamazoo, Michigan, USA.
[8]Lieber Institute for Brain Development, Johns Hopkins Medical Campus, Baltimore, Maryland, USA.
[9]Department of Psychiatry & Behavioral Sciences, Johns Hopkins School of Medicine, Baltimore, Maryland, USA.
[10]Department of Neurology, Johns Hopkins School of Medicine, Baltimore, Maryland, USA.
[11]Neuroscience Research Australia, Randwick, New South Wales, Australia.
[12]School of Medical Sciences, University of New South Wales, Kensington, New South Wales, Australia.
[13]Department of Laboratory Medicine and Pathobiology, University of Toronto, Toronto, Ontario, Canada.

Limited Dataset

	Features of CTE-NC (n=10)	Age	Suicide	Mood Disorder	Football	Contact Sports	TBI
1	No	75	No	No	No	No	Yes
2	No	75	No	No	No	No	No
3	No	62	No	No	No	No	No
4	No	51	No	No	No	No	Yes
5	No	64	No	Yes	No	No	No
6	No	72	No	No	No	No	Yes
7	No	68	No	Yes	No	No	No
8	No	63	No	No	No	No	No
9	No	75	No	No	No	No	No
10	No	75	No	Yes	No	No	No
11	No	72	No	No	No	No	No
12	No	63	No	Yes	No	Yes	No
13	No	91	No	No	Yes	Yes	No
14	No	84	No	Yes	Yes	Yes	.
15	No	61	No	Yes	No	Yes	No
16	No	58	No	No	Yes	Yes	No
17	No	67	No	No	No	No	No
18	No	84	No	Yes	No	No	Yes
19	No	51	No	Yes	No	No	No
20	No	51	No	No	No	No	No
21	No	50	No	Yes	No	No	No
22	No	54	No	Yes	Yes	Yes	Yes
23	No	67	No	Yes	No	No	No
24	No	78	No	No	No	No	No

	Features of CTE-NC (n=10)	Age	Suicide	Mood Disorder	Football	Contact Sports	TBI
25	No	61	No	Yes	Yes	Yes	No
26	No	57	No	Yes	Yes	Yes	Yes
27	No	69	No	No	No	No	No
28	No	52	No	No	No	No	.
29	No	54	No	No	Yes	Yes	No
30	No	55	No	No	Yes	Yes	No
31	No	95	No	Yes	No	No	No
32	No	69	No	No	No	No	No
33	No	58	No	Yes	No	No	No
34	No	84	No	No	No	No	No
35	No	61	No	Yes	No	No	No
36	No	60	No	No	No	No	Yes
37	No	61	No	Yes	No	Yes	Yes
38	No	63	No	No	No	No	No
39	No	51	No	Yes	Yes	Yes	No
40	No	56	No	Yes	Yes	Yes	Yes
41	No	61	No	Yes	No	No	Yes
42	No	71	No	No	No	No	No
43	No	51	No	No	No	No	No
44	No	60	No	No	Yes	Yes	Yes
45	No	52	No	No	No	No	No
46	No	75	No	No	No	No	No
47	No	66	No	Yes	Yes	Yes	.
48	No	66	No	No	No	No	.
49	No	52	No	Yes	Yes	Yes	Yes
50	No	76	No	Yes	Yes	Yes	No
51	No	62	No	Yes	Yes	Yes	Yes
52	No	59	No	Yes	Yes	Yes	Yes
53	No	74	No	No	No	No	No
54	No	52	No	No	No	No	No
55	No	79	No	No	Yes	Yes	No
56	No	71	No	No	No	No	No
57	No	55	No	No	No	No	No
58	No	82	No	No	No	No	No
59	No	52	No	No	Yes	Yes	Yes
60	No	57	No	No	No	No	No
61	No	52	No	Yes	No	No	Yes
62	No	59	No	No	No	No	No
63	No	53	No	No	No	No	No
64	No	64	No	No	Yes	Yes	No
65	No	81	No	No	Yes	Yes	No
66	No	69	No	Yes	No	No	No
67	No	64	No	No	No	No	No
68	No	69	No	No	No	No	Yes
69	No	54	No	No	No	Yes	No
70	No	70	No	No	No	No	No
71	No	65	No	Yes	No	No	No
72	No	80	No	Yes	Yes	Yes	Yes
73	No	51	No	No	No	No	Yes
74	No	83	No	No	No	No	Yes
75	No	67	No	No	No	No	No
76	No	66	No	Yes	No	No	No
77	No	65	No	No	No	No	.
78	No	80	No	No	No	No	Yes
79	No	69	No	No	Yes	Yes	No
80	No	59	No	No	Yes	Yes	No
81	No	69	No	Yes	No	No	No
82	No	96	No	No	No	No	No
83	No	68	No	No	No	No	No
84	No	66	No	Yes	No	No	No
85	No	59	No	Yes	Yes	Yes	.
86	No	54	No	No	Yes	Yes	Yes
87	No	60	No	Yes	Yes	Yes	Yes
88	No	67	No	Yes	Yes	Yes	Yes

	Features of CTE-NC (n=10)	Age	Suicide	Mood Disorder	Football	Contact Sports	TBI
89	No	73	No	No	Yes	Yes	No
90	No	80	No	No	No	No	No
91	No	70	No	No	No	Yes	Yes
92	No	61	No	No	No	No	No
93	No	55	No	Yes	No	No	No
94	No	70	No	No	No	No	No
95	No	62	No	No	No	No	No
96	No	59	No	Yes	No	No	Yes
97	No	77	No	No	No	No	No
98	No	71	No	No	No	No	No
99	No	75	No	No	No	Yes	No
100	No	66	No	Yes	Yes	Yes	.
101	No	56	No	Yes	No	No	Yes
102	No	61	No	Yes	No	No	No
103	No	60	No	Yes	No	No	No
104	No	64	No	Yes	No	No	No
105	No	69	No	Yes	No	No	No
106	No	65	No	Yes	No	No	No
107	No	72	No	No	No	No	Yes
108	No	63	No	Yes	No	No	No
109	No	50	No	No	Yes	Yes	Yes
110	No	75	No	No	No	No	No
111	No	52	No	Yes	No	No	No
112	No	84	No	No	No	No	No
113	No	69	No	No	Yes	Yes	No
114	No	68	Yes	No	No	No	No
115	No	57	Yes	Yes	No	No	No
116	No	77	Yes	Yes	No	No	No
117	No	79	Yes	Yes	No	No	No
118	No	78	Yes	Yes	No	No	No
119	No	51	Yes	No	No	No	Yes
120	No	74	Yes	Yes	Yes	Yes	No
121	No	59	Yes	Yes	No	No	No
122	No	64	Yes	Yes	Yes	Yes	Yes
123	No	93	Yes	No	No	No	No
124	No	79	Yes	Yes	No	No	No
125	No	67	Yes	Yes	No	No	No
126	No	62	Yes	Yes	No	No	Yes
127	No	62	Yes	Yes	No	No	Yes
128	No	51	Yes	Yes	No	No	No
129	No	60	Yes	No	No	No	Yes
130	No	83	Yes	Yes	No	No	No
131	No	60	Yes	Yes	No	No	No
132	No	80	Yes	No	No	No	No
133	No	53	Yes	Yes	No	Yes	Yes
134	No	66	Yes	Yes	No	No	No
135	No	66	Yes	Yes	Yes	Yes	No
136	No	54	Yes	No	No	No	Yes
137	No	89	Yes	Yes	No	No	Yes
138	No	73	Yes	No	No	No	No
139	No	74	Yes	No	No	No	No
140	No	52	Yes	Yes	No	No	Yes
141	No	78	Yes	Yes	No	No	Yes
142	No	62	Yes	No	No	No	No
143	No	89	Yes	Yes	No	No	No
144	No	62	Yes	Yes	No	No	No
145	No	65	Yes	Yes	No	No	Yes
146	No	87	Yes	Yes	No	No	No
147	No	55	Yes	Yes	No	No	No
148	No	52	Yes	Yes	Yes	Yes	No
149	No	68	Yes	Yes	No	No	No
150	No	57	Yes	Yes	No	No	Yes
151	No	60	Yes	Yes	Yes	Yes	Yes
152	No	54	Yes	Yes	No	No	Yes

	Features of CTE-NC (n=10)	Age	Suicide	Mood Disorder	Football	Contact Sports	TBI
153	No	57	Yes	Yes	No	No	No
154	No	54	Yes	Yes	Yes	Yes	Yes
155	No	69	Yes	Yes	No	No	No
156	No	61	Yes	Yes	No	No	No
157	No	78	Yes	Yes	No	Yes	Yes
158	No	70	Yes	Yes	No	No	No
159	No	75	Yes	Yes	No	No	No
160	No	52	Yes	Yes	No	No	No
161	No	65	Yes	Yes	No	No	No
162	No	79	Yes	Yes	Yes	Yes	No
163	No	74	Yes	Yes	Yes	Yes	Yes
164	No	56	Yes	No	No	No	No
165	No	57	Yes	Yes	Yes	Yes	No
166	No	89	Yes	Yes	Yes	Yes	No
167	No	61	Yes	Yes	No	No	No
168	No	72	Yes	Yes	No	No	No
169	No	72	Yes	Yes	Yes	Yes	No
170	No	51	Yes	Yes	No	No	No
171	No	50	Yes	Yes	No	Yes	No
172	No	51	Yes	Yes	No	No	No
173	No	61	Yes	Yes	No	No	No
174	No	85	Yes	Yes	Yes	Yes	No
175	No	57	Yes	No	No	No	Yes
176	No	51	Yes	Yes	Yes	Yes	Yes
177	Yes	92	No	No	No	No	No
178	Yes	75	No	No	Yes	Yes	Yes
179	Yes	75	No	No	No	No	No
180	Yes	77	No	No	Yes	Yes	No
181	Yes	52	No	Yes	Yes	Yes	No
182	Yes	91	No	No	Yes	Yes	No
183	Yes	89	Yes	Yes	No	No	No
184	Yes	54	Yes	Yes	No	No	Yes
185	Yes	51	Yes	Yes	No	Yes	No
186	Yes	83	Yes	Yes	No	No	No
Total	186	186	186	186	186	186	179
Positive Responses	10	--	67	103	48	58	50

Note: CTE-NC=Chronic traumatic encephalopathy neuropathologic change (possible features); Mood disorder=Depression or Bipolar Disorder; and TBI=Traumatic brain injury.
